# Protein Valuation in Food Choice Is Positively Associated with Lean Mass in Older Adults

**DOI:** 10.1093/jn/nxz124

**Published:** 2019-06-14

**Authors:** Charlotte M Buckley, Sophie Austin, Bernard M Corfe, Mark A Green, Alexandra M Johnstone, Emma J Stevenson, Elizabeth A Williams, Jeffrey M Brunstrom

**Affiliations:** 1 Nutrition and Behaviour Unit, School of Psychological Science, University of Bristol, Bristol, United Kingdom; 2 Department of Oncology and Metabolism, The Medical School, The University of Sheffield, Sheffield, United Kingdom; 3 Department of Geography and Planning, School of Environmental Sciences, University of Liverpool, Liverpool, United Kingdom; 4 Rowett Institute, School of Medicine, Medical Sciences and Nutrition, University of Aberdeen, Aberdeen, United Kingdom; 5 Human Nutrition Research Centre, Institute of Cellular Medicine, Medical School, Newcastle University, Newcastle upon Tyne, United Kingdom; 6 National Institute for Health Research, Bristol Biomedical Research Centre, University Hospitals Bristol NHS Foundation Trust and University of Bristol, Bristol, United Kingdom

**Keywords:** protein valuation, sarcopenia, food choice, body composition, fat-free mass index, lean mass, aging

## Abstract

**Background:**

Calorie for calorie, protein is more satiating than carbohydrate or fat. However, it remains unclear whether humans perceive calories derived from these macronutrients equally and whether lean mass is associated with a tendency to “value” protein when dietary decisions are made.

**Objectives:**

This study aimed to determine the test-retest reliability of a novel method for quantifying macronutrient valuations in human volunteers and to determine whether “protein valuation” is associated with a higher fat-free mass index (FFMI) in older adults.

**Methods:**

A 2-alternative, forced-choice task in which 25 foods were compared in 300 trials was undertaken in 2 studies. In study 1, participants (age range 19–71 y, *n* = 92) attended 2 test sessions, spaced 1 wk apart. In study 2, older adults (age range 40–85 y; *n* = 91) completed the food-choice task and assessed the test foods for liking, expected satiety, and perceived healthiness. Body composition and habitual protein intake were assessed in both studies. Data were analyzed through the use of individual binomial logistic regressions and multilevel binomial logistic regressions.

**Results:**

In study 1, measures of macronutrient valuation showed excellent test-retest reliability; responses in the forced-choice task were highly correlated (week 1 compared with week 2; protein, *r* = 0.83, *P* < 0.001; carbohydrate, *r* = 0.90, *P* < 0.001; fat, *r* = 0.90, *P* < 0.001). Calorie for calorie, protein and carbohydrate were stronger predictors of choice than fat (*P* < 0.001). In study 2, protein was a stronger predictor than both carbohydrate (*P* = 0.039) and fat (*P* = 0.003), and a positive interaction was observed between protein valuation and FFMI (OR = 1.64; 95% CI: 1.38, 1.95; *P* < 0.001). This was the case after controlling for age, gender, liking for foods, and habitual protein consumption.

**Conclusions:**

Together, these findings demonstrate that adult humans value calories derived from protein, carbohydrate, and fat differently, and that the tendency to value protein is associated with greater lean mass in older adults.

## Introduction

Many modern foods are energy dense (kcal/g) and the role this plays in promoting energy intake has been explored extensively (1). Measures of energy density are useful because they provide a guide to the total energy in a fixed portion of food. However, humans do not detect energy density directly and foods with equal energy density might differ in their fat, protein, and carbohydrate content, each of which is absorbed and utilized in different ways (2).

Studies have considered how chronically high intakes of fats and carbohydrates can promote obesity and cardiometabolic disease (3, 4). Conversely, lower intake of protein is a risk factor for sarcopenia—an age-associated decline of skeletal muscle tissue that can influence physical functioning and quality of life (5–7). Intervention studies have demonstrated a causal association: muscle function is impaired when protein intake is reduced (8) and protein supplementation produces a corresponding improvement (9).

Additionally, variation in chronic macronutrient intake is considerable (10) and is influenced by individual dietary decisions, which, in turn, are governed by environmental (e.g., food availability and cost) and subjective factors (e.g., expected satiety and perceived healthiness). However, as with other omnivores, humans also have an inherent ability to discriminate foods based on their macronutrient composition and do so through the use of both sensory information (11, 12) and via learning (13). For example, low-protein diets promote the ingestion of savory high-protein foods (14, 15), and sweet tastes (related to carbohydrate) are selected after physical activity (16). These observations imply that acute changes in physiologic state can affect the way that humans value and prioritize energy derived from different macronutrients.

This distinction between habitual macronutrient consumption (typically measured through the use of an FFQ) and macronutrient valuation (an underlying disposition to select foods according to how macronutrients are prioritized) is important. For example, an individual's selection of a fried breakfast over oatmeal might reflect high fat valuation, or it might otherwise reflect habit or a general desire for a larger meal (the absolute difference in fat might be incidental). Instead, high fat valuation would be evidenced when fat influences choice even when foods with almost identical amounts of fat are compared—a small difference plays a role because calories from fat are still “noticed” and influence choice. Similarly, a person with high carbohydrate valuation would be sensitive to small differences in carbohydrate and would select the more carbohydrate rich of 2 foods even when low-carbohydrate-containing foods are compared.

Here, we describe a novel approach that enables researchers to quantify underlying macronutrient valuations. After controlling for expected satiety and perceived healthiness, we then used this approach to explore individual differences. Specifically, in a second study we predicted that people with high protein valuation will have greater fat-free mass and explored this relation in a group of older adults.

## Methods

### Study objectives

Study 1 sought to investigate the test-retest reliability of our measure of macronutrient valuation and to quantify differences in the valuation of fat, carbohydrate, and protein. Study 2 aimed to explore the relation between protein valuation and fat-free mass in a group of older participants.

### Study 1

#### Subjects

Ninety-two participants were recruited into the study. This was based on an earlier unpublished study which observed a small-to-medium effect size (*r* = 0.3) of macronutrient valuation in food choice (17). We determined that a minimum sample size of 90 participants would be required with an α of 0.05 (18). Participants were recruited from the population of staff and students at the University of Bristol, UK and from the surrounding area via an existing volunteer database and newspaper advertisements. To enable participants to complete the food-choice measures, they were required to have English as a first language or an equivalent level of fluency, and were excluded if they were vegan or vegetarian, or if they reported a food allergy or intolerance.

#### Procedure

An online questionnaire was used to collect demographic information (age, gender, and postcode) and responses to the Dutch Eating Behavior Questionnaire (DEBQ) (19). On a separate day, participants attended the Nutrition and Behavior Unit, University of Bristol for the first of 2 test sessions, held at the same time of day and 1 wk apart. Each session lasted ∼30 min and they were scheduled at the same time of day between 0900 and 1700. On arrival, participants read an information sheet and signed a consent form. They then completed the 2-alternative forced-choice task, followed by measures of expected satiety, liking, perceived healthiness, and familiarity. At the end of the second test session body weight and height were measured according to standardized protocols. Participants were then debriefed and offered £15 in remuneration for their assistance.

### Study 2

#### Subjects

Participants completed either an online questionnaire or a short telephone interview to confirm eligibility. The same exclusion criteria were applied as in study 1. However, participants were also excluded if they were pregnant or breastfeeding, had diabetes, were taking any medications that might affect their appetite, had recently started taking a medication, were undergoing hospital treatment, had a significant current or past psychiatric illness (including Alzheimer's and dementia), or had a current or previous eating disorder. After screening, 91 participants were invited to attend the Nutrition and Behavior Unit for a single session that was scheduled between 0900 and 1700 and that lasted ∼90 min.

#### Method

The beginning of the test session was identical to study 1. However, after the computer-based measures and the DEBQ, participants also completed an FFQ. The online FFQ comprised 149 items and was based on the European Prospective Investigation into Cancer and Nutrition (EPIC) (20)—a version modified to include wholegrain and to assess intake over 7 d. The FFQ was automated to analyze the nutritional composition of the diet and provided an estimate of the proportion (%) of dietary energy intake derived from protein. Gender, postcode, and height were recorded, and bioelectrical impedance analysis (BC-418 MA III Body Composition Analyzer; Tanita Corporation) was used to measure body mass, fat mass, and fat-free mass. Measures of BMI (kg/m^2^), body-fat percentage, and fat-free mass index (FFMI, kg/m^2^) were derived from these data. The FFMI was calculated by dividing fat-free mass by height squared (21). At the end of the session, participants were debriefed and offered £15 in remuneration for their assistance.

### Ethics

Both studies were conducted according to the ethical guidelines laid down in the Declaration of Helsinki and were approved by the University of Bristol Science Faculty Ethics Committee (approval codes: study 1: 52163, study 2: 59121). Written informed consent was obtained from all participants. The aims and objectives of both studies were preregistered on the Open Science Framework (17, 22). In each case, this incorporated preplanned hypotheses as outlined in the introduction. No participant took part in both studies.

### Food evaluation tasks

Images were taken of 25 different foods in 100-g portions. In a computerized 2-alternative forced-choice task, images of 2 different foods were presented side-by-side on a computer screen. Every combination of food pairings was presented, rendering 300 binary-choice trials. The order of the trials was randomized (separately for each participant) and in each trial the relative position of each food (left or right) was allocated randomly. Participants were given the following instruction: “You will be shown two picnic foods, imagine this will be the only food you can eat between breakfast at 9am and dinner at 7pm and you must only pick one of the two foods.” Stimuli were carefully selected to include a range of foods that varied in macronutrient composition and to minimize intercorrelations between sources of protein, fat, and carbohydrate. Correlations (Pearson *r*) between calories derived from fat and protein, fat and carbohydrate, and protein and carbohydrate in the food images were 0.28, −0.33, and −0.36, respectively. Stimuli were also selected because they are referenced as foods that are commonly consumed in the United Kingdom (23). **Table 1** includes a description of each food, together with its nutritional composition.

**TABLE 1:** Nutritional composition of test foods in study 1 and 2

		kcal/100 g		
Food	Energy density, kcal/g	Protein	Carbohydrate	Fat
Apple	0.5	2.0	48.0	4.5
Avocado	2.0	7.6	7.6	175.5
Bacon	2.3	103.2	4.0	124.2
Bagel	2.6	41.2	195.6	11.7
Baked beans	0.8	18.8	51.6	1.8
Banana	1.0	4.8	92.0	4.5
Blueberries	0.5	3.6	36.4	4.5
Broccoli	0.4	17.2	12.4	5.4
Cheddar cheese	3.3	113.2	8.4	203.4
Chicken	1.1	95.6	2.0	14.4
Coleslaw	1.8	3.2	21.6	153.0
Crumpets	2.1	26.4	170.4	9.9
Egg	1.4	56.4	2.0	86.4
Grapes	0.7	2.0	61.6	4.5
Ham	1.1	76.0	6.8	20.7
Mediterranean vegetables	0.6	4.4	31.6	15.3
Mushrooms	0.2	7.2	2.0	4.5
Pasta	1.6	20.4	130	6.3
Potato salad	1.4	4.0	42.4	91.8
Potato waffle	1.8	10.0	88.0	78.3
Prawns	0.6	56.4	2.0	4.5
Sausage	2.5	52.0	26.8	168.3
Smoked salmon	1.9	80.4	13.2	92.7
Sweet potato	0.9	4.4	75.6	4.5
Tuna	1.1	108	2.0	4.5

Expected satiety was measured by presenting an image of each test food alongside an image of a plate of rice. The portion of rice ranged in 20-kcal increments (20–800 kcal) and participants adjusted the portion of rice until they were confident that both portions would reduce their hunger for the same amount of time. This and all other tasks were implemented with the use of custom software written in Visual Basic (freely available on request).

Visual analog scales (VASs) were used to elicit ratings of healthiness and liking. For healthiness, the VAS was headed “How healthy is this food?” and anchored with “Not at all healthy” and “Extremely healthy”. For liking, the VAS was headed “How much do you like the taste of this food?” and anchored with “I hate it” and “I love it.” In both cases, responses were assigned a value in the range 0 to 100. To assess familiarity, participants responded to the question “Have you eaten this food before?” with response options “yes” or “no.”

In measures of expected satiety, liking, healthiness, and familiarity, each food was presented in turn and in a random order. The DEBQ (19) was used to characterize trait dietary styles in our samples. Separate subscales assess restrained, emotional, and external eating. Participant postcodes were recorded, which were used to estimate “neighborhood deprivation”, a proxy for socioeconomic status (24).

### Statistical analysis

All statistical analyses were conducted in the R environment (25) with the use of the lme4 add-on package (26), and figures were created with the ggplot2 add-on package (27).

### Valuation of individual macronutrients

In study 1, 8 participants did not attend both sessions and were not included in the final analysis; overall, 57 females and 27 males completed both test sessions. In study 2, data from 1 participant were excluded because of a computer error. Therefore, data from 23 males and 68 females were analyzed. When a participant was unfamiliar with 1 of the foods, then data from any associated trial were removed. On this basis, we excluded 3121 (6.6%) trials in study 1 and 998 (4.2%) trials in study 2.

In the 2-alternative forced-choice task, for each participant and each trial, an “energy-density difference score” was computed by subtracting the energy density (kcal/g) of the food presented on the left from the energy density of the food on the right. Separate difference scores were also calculated for calories derived from protein, fat, and carbohydrate, and for differences in expected satiety (kcal) and healthiness (mm). To enable direct comparison between expected satiety and healthiness, difference scores for these predictors were standardized within each participant.

We used binary logistic regression to enter energy-density difference scores as predictors of choice. For study 1, a separate model was computed for each participant and each test session (84 × 2 models). For study 2, a single model was computed for each participant (91 models). We used the same approach to also generate models by entering differences in protein, carbohydrate, fat, expected satiety, and healthiness, as simultaneous predictors of choice (259 models). In each model, every β coefficient was exponentiated to produce an OR—an unbiased estimate of the relative contribution of each predictor as a determinant of choice. A protein OR refers to the odds of choosing the left-hand food when the left-hand food contains 1 kcal/g more protein than the right-hand food. Similarly, an OR for carbohydrate or fat can be interpreted in the same way. Importantly, these 3 ORs quantify each macronutrient valuation. To determine whether fat, protein, and carbohydrate differ in valuation, a 1-way ANOVA was used, with macronutrient type (protein, carbohydrate, and fat) as a predictor of OR. Tukey-adjusted post-hoc tests were used to explore differences between individual macronutrients, and *t* tests were used to determine whether sets of ORs deviate from 1.0 (evidence that choice is influenced by a predictor).

### Test-retest reliability

For study 1, test-retest reliability was assessed by evaluating the association between participant ORs for the 2 test sessions. Separate Pearson's coefficients were computed for energy density and for each macronutrient.

### Relation between FFMI and protein valuation

Due to machine error, fat-free mass was not recorded for 7 participants and their data were excluded from this analysis. The remaining data comprised 24,201 trials from 84 participants. To account for the intraclass correlation between individual participant responses (28) a multilevel (rather than a standard generalized linear model) binary-logistic modeling approach was adopted.

#### Basic model

For study 2, our objective was to explore the extent to which protein valuation is associated with a higher FFMI. For each trial, the difference between the protein content (standardized kcal/g) of the 2 foods was entered as a predictor of choice. The associated OR from the model provides a measure of protein valuation across participants. We also specified the interaction between protein difference and FFMI. A positive interaction indicates that protein valuation is stronger in participants with greater muscle mass. In addition, “participant” was entered as a random factor, and age and gender were included as covariates.

#### Extended model

In an extended model, we specified an identical model that also included liking difference scores and their interaction with protein-difference scores. The model also incorporated habitual protein intake (% energy in diet) and the interaction between habitual protein intake and protein-difference scores. Note that the main effect of habitual protein intake was not expected to predict choice (i.e., make participants preferentially choose the option presented on the left), but was included to properly assess the interaction with protein difference. As above, a positive interaction between FFMI and protein difference indicates that protein valuation is stronger in individuals with a higher fat-free mass, and that this is independent of liking for high-protein foods or habitual protein consumption. For this model, 1 participant was excluded due to missing responses on the FFQ. An exploratory analysis was also conduced, extending the extended model to add an interaction term between gender, protein difference, and FFMI.

For both multilevel models, ORs, CIs, and *P* values are reported, and a main or interaction effect was regarded as a significant predictor of choice if the 95% CI for an OR failed to cross 1.0. To enable a direct comparison of their relative importance, all variables were standardized before entering them into the food-choice models. Unless specified otherwise, data are presented as means ± SDs.

## Results

### Participant demographics

Ninety-two participants completed study 1 (68% female). Their ages ranged from 19 to 71 y (24.9 ± 7.30 y) and their mean BMI was 23.0 ± 3.9. Ninety-one participants completed study 2 (75% female). They had mean BMI of 26.2 ± 4.3 and their ages ranged from aged 40 to 85 y (60.6 ± 12.2 y). **Table 2** provides additional demographic information about the participants in both studies.

**TABLE 2:** Participant demographic information for men and women aged 19–85 y in study 1 and 2^1^

	Study 1. *n* = 84 (female = 57)	Study 2. *n* = 91 (female = 68)
% female	67.8	74.7
Age, y	25.1 ± 8.4 (19–71)	60.6 ± 12.3 (40–85)
BMI, kg/m^2^	23.0 ± 4.2 (14.7–29.7)	26.2 ± 4.3 (18.2–37.9)
FFMI, kg/m^2^	—	17.3 ± 2.5 (12.4–24.3)
Habitual protein consumption, % of total energy	—	14.3 ± 2.5 (9.1–21.5)
Index of multiple deprivation	14.2 ± 9.3 (2.6–46.7)	16.7 ± 12.1 (2.6–53.3)
DEBQ emotional	2.4 ± 0.8 (1.0–4.6)	2.2 ± 0.8 (1.0–3.8)
DEBQ external	3.3 ± 0.6 (1.9–3.9)	3.0 ± 06 (1.3–4.9)
DEBQ restraint	2.4 ± 0.7 (1.0–3.9)	2.9 ± 0.8 (1.1–4.8)

1 Values are means ± SDs (ranges) or percentages. DEBQ, Dutch Eating Behavior Questionnaire; FFMI, fat-free mass index.

### Test-retest reliability

**FIGURE 1:**
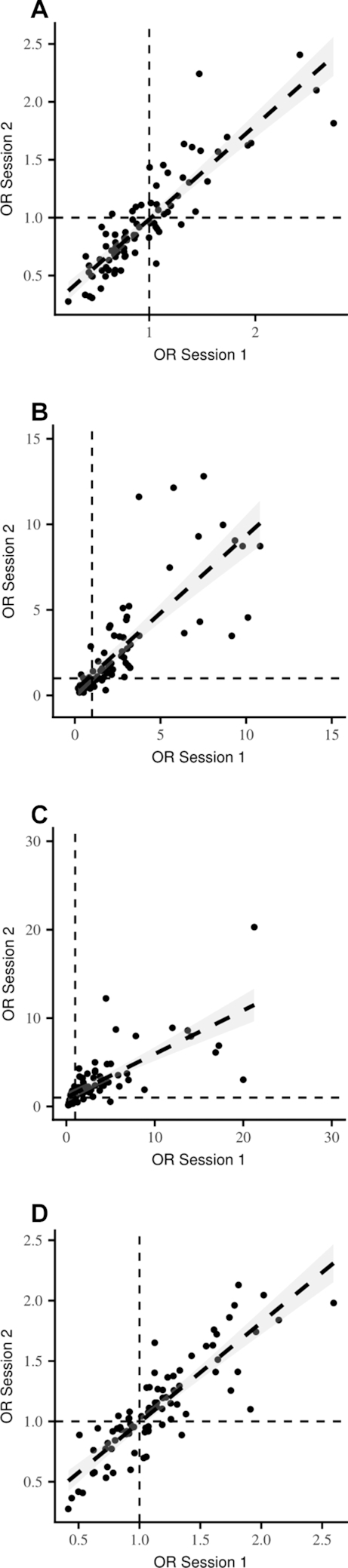
Relations between ORs obtained for men and women aged 19–71 y in session 1 and 2 in study 1. Panels show associations for (A) fat, (B) carbohydrate, (C) protein, and (D) energy. Note: short dashed lines represent OR = 1 (no significant effect on food choice). Long dashed lines show the correlation (shaded ±1 95% CI) between participant ORs between sessions. Each data point shows 2 ORs, obtained from a single participant (total n = 84) tested on 2 occasions, separated by a 1-wk interval.

**Figure 1** shows relations between ORs obtained from separate participants in session 1 and session 2 in study 1. Figure 1A, B, C, and D show associations for fat, carbohydrate, protein, and overall energy density, respectively. In each case, we observed strong positive relations, indicating excellent test-retest reliability across sessions: protein, *r* = 0.71, *P* < 0.001; carbohydrate, *r* = 0.97, *P* < 0.001; fat, *r* = 0.90, *P* < 0.001; energy, *r* = 0.86, *P* < 0.001. Inspection of Figure 1 also shows considerable individual variability in the relative importance of food characteristics as predictors of choice and that this variability is quite stable over a 1-wk period.

### Liking and familiarity

The foods were well liked (study 1: 67.9 ± 10.6 mm, range = 39–93 mm; study 2: 66.1 ± 11.2 mm, range = 42–77 mm) and familiar (study 1, 96.5% ± 4.1%, range = 82–100%; study 2, 98% ± 2.8%, range = 88–100%). **Table 3** shows descriptive statistics (means and SDs) for individual foods and for each study, separately.

**TABLE 3:** Ratings for liking, healthiness, expected satiety, and familiarity from men and women aged 19–85 y in study 1 and 2^1^

	Study 1				Study 2			
Food	Liking	Healthiness	Expected satiety	Familiarity	Liking	Healthiness	Expected satiety	Familiarity
Apple	74.5 ± 18.8	87.6 ± 10.5	146.8 ± 109.8	100	78.6 ± 19.4	90.1 ± 9.7	183.3 ± 108.5	100
Avocado	63.2 ± 30.9	83.6 ± 13.0	198.1 ± 107.3	94	66.2 ± 32.1	80.1 ± 19.2	226.2 ± 105.4	98
Bacon	71.5 ± 25.9	16.3 ± 17.4	282.4 ± 128.4	98	70.9 ± 24.7	23.6 ± 19.5	302.6 ± 133.9	100
Bagel	68.8 ± 21.3	32.1 ± 16.9	266.4 ± 131.3	98	48.1 ± 25.8	32.5 ± 17.0	264.0 ± 120.1	92
Baked beans	55.5 ± 26.1	48.6 ± 21.3	178.6 ± 118.4	95	62.9 ± 24.1	65.9 ± 20.2	216.0 ± 108.5	100
Banana	92.7 ± 18.5	85.7 ± 12.7	165.4 ± 86.9	99	77.8 ± 22.9	83.8 ± 13.9	223.7 ± 93.7	99
Blueberries	75.3 ± 22.3	90.9 ± 11.9	142.4 ± 102.5	98	74.6 ± 26.0	90.9 ± 10.3	174.1 ± 100.8	97
Broccoli	65.6 ± 26.2	95.0 ± 6.2	137.4 ± 73.1	98	68.0 ± 26.2	91.3 ± 8.7	173.4 ± 98.0	97
Cheddar cheese	73.1 ± 23.9	30.9 ± 19.2	227.1 ± 113.7	100	77.1 ± 21.1	45.6 ± 21.4	293.6 ± 139.6	99
Chicken	77.3 ± 17.1	71.7 ± 18.8	218.8 ± 119.7	99	79.9 ± 17.2	77.1 ± 16.7	255.4 ± 110.8	100
Coleslaw	38.8 ± 29.7	30.9 ± 19.2	148.7 ± 113.4	82	48.3 ± 26.9	40.0 ± 19.5	178.7 ± 92.1	97
Crumpets	66.1 ± 22.2	30.5 ± 18.1	239.5 ± 98.6	92	60.2 ± 25.3	28.7 ± 15.9	257.4 ± 119.7	100
Egg	66.5 ± 26.9	76.4 ± 14.6	183.5 ± 90.8	99	71.4 ± 24.6	75.5 ± 15.9	210.5 ± 100.9	100
Grapes	81.1 ± 18.3	83.7 ± 15.3	99.8 ± 75.9	99	82.6 ± 15.3	85.2 ± 13.4	150.3 ± 127.8	100
Ham	54.8 ± 24.2	38.1 ± 24.9	200.5 ± 98.7	99	58.7 ± 24.5	32.6 ± 22.2	260.4 ± 123.5	100
Mushrooms	63.4 ± 27.3	83.8 ± 14.0	142.4 ± 103.2	99	74.2 ± 23.9	80.5 ± 16.7	177.8 ± 125.8	99
Pasta	70.1 ± 21.9	44.7 ± 17.9	231.3 ± 105.1	100	59.5 ± 23.8	52.2 ± 19.7	240.2 ± 101.3	98
Potato salad	52.9 ± 24.9	32.7 ± 17.8	169.2 ± 91.2	95	48.1 ± 27.0	37.2 ± 16.5	184.4 ± 84.7	97
Prawns	63.4 ± 25.8	21.1 ± 13.9	214.4 ± 96.7	90	68.5 ± 28.5	73.3 ± 18.8	215.4 ± 97.2	97
Potato waffle	67.4 ± 26.7	70.1 ± 17.3	193.2 ± 79.7	96	42.3 ± 28.9	20.0 ± 14.9	239.8 ± 108.1	88
Sausage	69.3 ± 25.0	20.9 ± 15.4	218.8 ± 98.6	100	57.6 ± 28.2	21.0 ± 17.1	240.7 ± 108.9	99
Smoked salmon	73.6 ± 25.1	71.0 ± 17.0	204.7 ± 106.9	94	67.4 ± 31.8	69.6 ± 20.5	221.5 ± 121.8	96
Sweet potato	77.9 ± 19.5	74.0 ± 18.0	232.0 ± 95.3	96	67.4 ± 27.2	79.9 ± 16.0	239.8 ± 89.0	99
Tuna	60.7 ± 26.0	73.4 ± 16.8	211.3 ± 95.1	94	61.5 ± 29.8	76.9 ± 19.4	249.7 ± 111.6	96
Vegetables	74.4 ± 20.7	83.4 ± 13.7	149.9 ± 82.0	99	79.7 ± 20.5	81.3 ± 15.1	194.7 ± 85.2	100

1 Values are means ± SDs or percentages, liking and healthiness are measured on a 0–100 scale, and expected satiety is measured in kcal. Familiarity is the proportion (%) of participants who indicated they were familiar with the food.

### Energy density as a predictor of choice

In study 1, as anticipated, energy density was a positive predictor of choice in both session 1 (OR = 1.08; 95% CI: 1.06, 1.10; *P* < 0.001) and session 2 (OR = 1.05; 95% CI: 1.03, 1.07; *P* < 0.001). These ORs show that when 2 foods differ in energy density by 1 kcal/g, then the more energy-dense food was 8% more likely to be selected in session 1 and 5% more likely to be chosen in session 2. These effects are small, but statistically significant. By contrast, energy density (kcal/g) was a nonsignificant predictor (OR did not deviate from 1.0) of choice in study 2 (OR = 1.02; 95% CI: 0.93, 1.11; *P =* 0.642).

### Individual macronutrients and psychological variables as predictors of choice

**FIGURE 2:**
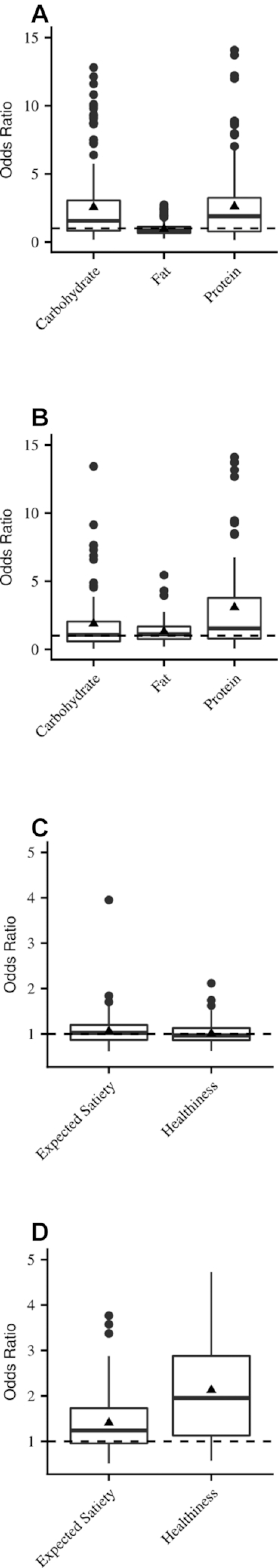
Box and whisker plots describing ORs for predictors of choice for men and women aged 19–81 y in study 1 and study 2. Separate panels show ORs for macronutrients in study 1 (A) and study 2 (B) and psychological predictors (expected satiety and healthiness) for study 1 (C) and study 2 (D). Note: dashed line indicates no effect on choice. In cases where a 95% CI fails to cross this line, then the associated variable has a nonrandom effect on choice. ORs for study 1 were averaged across test sessions. For all figures, the black triangle indicates mean OR, the black line represents the median, the upper edge of the box represents the 75% quartile, and the lower edge represents the 25% quartile. Black dots represent outliers.

**Figure 2** shows the extent to which protein, carbohydrate, fat, expected satiety, and healthiness played a role in food choice. In each case, separate ORs are provided for study 1 and study 2. Because we observed very good test-retest reliability (see Figure 1), for each participant, we averaged separate ORs across sessions in study 1. ORs for protein, carbohydrate, and expected satiety (but not fat or healthiness) were significantly larger than 1, suggesting they independently influence food choice. Associated statistics are summarized in **Supplemental Table 1**. One-way ANOVA confirmed that average odds ratios also differed across macronutrients, *P* < 0.001. Tukey-adjusted post-hoc tests showed that carbohydrate (*P* < 0.001) and protein (*P* < 0.001) were stronger predictors of choice than fat. There was no difference in ORs for protein compared with carbohydrate (*P =* 0.775) and the difference between expected satiety and healthiness was marginal (*P =* 0.055).

Mean ORs for protein, carbohydrate, and fat also differed in study 2, *P =* 0.003. Tukey-adjusted post-hoc tests demonstrated that protein had a stronger influence on choice than carbohydrate (*P* = 0.039) or fat (*P* = 0.003), and there was no difference between ORs for carbohydrate compared with fat (*P* = 0.654). ORs for carbohydrate and fat did not differ (*P =* 0.654) and healthiness was a stronger predictor of choice than expected satiety (*P* < 0.001).

### How do individual differences in protein valuation interact with body composition to predict choice?

**Tables 4** and **5** summarize the basic and extended models used to explore the interaction between body composition and protein valuation. The basic model showed a positive interaction between protein valuation and FFMI as a predictor of food choice. A difference in protein (kcal/g) is a stronger predictor of choice in individuals with a higher FFMI, after controlling for age and gender (*P* < 0.001). The extended model indicates that this interaction is also observed after controlling for liking and habitual protein consumption. In this model, for an individual with a higher FFMI (+1 SD), a 1-kcal/g (standardized) difference in protein content is associated with increased odds of 64% of choosing that food (*P* < 0.001).

**TABLE 4:** Summary of fixed parts of 2 hierarchical multilevel binomial logistic regressions predicting food choice from protein content, FFMI, habitual protein consumption, and liking for men and women aged 19–85 y in study 1 and 2^1^

	Basic model		Extended model	
Fixed parts	OR (95% CI)	*P*	OR (95% CI)	*P*
Intercept	1.02 (0.87,1.20)	0.780	0.97 (0.79,1.19)	0.775
Protein difference	1.13 (0.99,1.30)	0.790	0.86 (0.72,1.02)	0.075
Protein difference × FFMI	1.47 (1.28, 1.70)	<0.001	1.64 (1.38,1.95)	<0.001
Protein difference × age	1.00 (1.00,1.00)	0.815	1.00 (1.00,1.01)	<0.001
Protein difference × gender^2^	1.26 (1.15,1.39)	<0.001	1.37 (1.22,1.53)	<0.001
FFMI × gender^2^	1.03 (0.94,1.12)	0.518	1.09 (0.98, 1.22)	0.100
Protein difference × FFMI × age	1.00 (0.99,1.00)	0.001	0.99 (0.99, 1.00)	<0.001
Protein difference × FFMI × gender^2^	0.84 (0.78,0.90)	0.001	0.78 (0.71,0.85)	<0.001
Liking difference	—	—	5.54 (5.28,5.82)	<0.001
Protein difference × habitual protein consumption %	—	—	1.02 (0.99,1.05)	0.214
Protein difference × liking difference	—	—	1.05 (1.00,1.10)	0.065

1 Age and gender are covariates in the model. In a separate analysis, carbohydrate and fat difference scores were added to the extended model as an interaction term with FFMI. Carbohydrate difference negatively interacted with FFMI to predict food choice (OR = 0.86; 95% CI: 0.83, 0.91; *P* < 0.001) and fat difference did not interact with FFMI to predict food choice (OR = 1.00; 95% CI: 0.97, 1.04; *P =* 0.901). FFMI, fat-free mass index.

2 Reference group = female.

**TABLE 5:** Summary of random parts of 2 hierarchical multilevel binomial logistic regressions predicting food choice^1^

Random parts	Basic model	Extended model
τ_00, Participant_	0.005	0.009
N_Participant_	84	83
ICC_Participant_	0.002	0.003
Observations	24,201	23,925
Tjur's *d*	0.010	0.320
Deviance	33,269.496	24,258.752

1 ICC, intraclass correlation.

## Discussion

Numerous studies have explored the relation between food energy density, food intake, and food preference (29). Here, we introduce a novel method that quantifies the underlying value that humans place on a calorie derived from fat, carbohydrate, and protein. Study 1 shows that protein and carbohydrate tend to be valued more than fat (compared calorie for calorie). However, we also observed considerable variability across individuals. Indeed, these differences showed excellent test-retest reliability across 2 sessions, held 1 wk apart. In study 2, protein was valued more than carbohydrate and fat and, again, we observed the same variability across individuals. In study 2 we also found that individuals with a higher FFMI show greater protein valuation. Because body composition can be influenced by protein consumption (8), this correspondence with protein valuation further validates our approach.

Note that the relation between FFMI and protein valuation was observed after controlling for age, gender, liking for foods, and habitual protein consumption. In other words, protein valuation appears to be associated with FFMI and this occurs even after controlling for an estimate of protein consumption obtained from a widely used FFQ. Following other work (30) we transformed the OR (1.64) for this interaction term into a Cohen's *d*. The associated effect size (*d* = 0.27) indicates that the effect of differences in protein valuation is small but could be important at a population level.

In relation to the above, the interaction between habitual protein consumption (measured by FFQ) and “protein difference” (Table 4) also merits careful consideration. A significant interaction would indicate that people who report consuming a high-protein diet are especially sensitive to small differences in the protein content of food pairs in the choice task, and selected foods on this basis. This interaction was not observed, suggesting that protein valuation is not governed exclusively by differences in self-reported protein intake. Again, to clarify this distinction, protein valuation refers to an underlying sensitivity to small differences in protein, which biases *all* food choices. By contrast, FFQs provide an estimate of habitual protein intake, which, in turn, will also be governed by cost, availability, liking, and so on (31). This distinction between protein valuation and habitual protein intake is important—high valuation will promote greater protein intake, but this relation is not axiomatic—a person might have high valuation, but low protein intake due to food availability. Conversely, a high protein intake might be reported (perhaps governed by family shopping habits) even in someone with low valuation. In other words, there might be multiple interacting determinants of total protein intake including both opportunity (the environment) and valuation.

The temporal direction of the association between protein valuation and FFMI is currently unclear. One possibility is that muscle mass plays a causal role in food choice—higher protein valuation reflects a bias that serves to ensure that a biologically determined amount of muscle mass is preserved. Alternatively, differences in protein valuation may occur for other reasons and, over the life span, they have a secondary and incidental effect on muscle mass. We suspect the latter is more likely because there is little evidence that sarcopenia is associated with an increased preference for protein (indeed, the converse seems more likely) (7). Indeed, individuals with reduced protein valuation may be particularly vulnerable to sarcopenia as they age. If correct, then our methods might be applied to identify individuals for targeted dietary advice, before age-related muscle deterioration occurs. This is important because a 30–40% decrease in muscle mass occurs between the ages of 40 and 80 y (32), which suggests that interventions should occur in the fourth decade of life (6).

In future, studies might incorporate a measure of physical activity, which is known to influence muscle synthesis after protein intake (33), and a major factor influencing muscle wastage is low physical activity during aging (34). Indeed, the combined effects of low protein valuation and low physical activity might place an individual at an even greater risk of muscle loss with aging. Other determinants of protein intake such as socioeconomic status may be used alongside our method to clarify the relation between protein valuation, protein intake, and physical activity, particularly as risk factors for protein undernutrition and sarcopenia. A second future question relates to whether humans discriminate the protein quality (amino acid profile) of different sources of protein and show differential protein valuation on this basis. Animal- and plant-based sources might be compared, addressing both fundamental questions and broader concerns about food security, the environment, and health (6).

We also observed a 3-way interaction between gender, FFMI, and difference in protein content. This was not an a priori prediction and therefore the study was not powered to investigate gender-related differences in protein valuation and their relation to FFMI. However, it is worth noting that sarcopenia develops at a different rate in men and women (35). In our healthy community-dwelling sample, we saw little evidence that protein valuation changes markedly with age. Again, in an appropriately powered sample this might be investigated. A further step would be to administer this task to people with sarcopenia to test the prediction that extreme muscle deterioration is associated with especially low protein valuation. Note that although FFMI has been used to assess sarcopenia previously (36), assessments of muscle strength might also be incorporated in this context.

In addition, we see opportunities to apply our methods to address fundamental questions about human appetite control. Various sources indicate that omnivores adapt their dietary behavior in response to periods consuming a low-protein diet (37). Some indicate a strategic orientation towards high-protein foods (14, 15) and others suggest a more general adaptation whereby overall intake is increased to mitigate a shortfall in protein (38). However, in both cases the evidence is mixed (39) and is limited in humans. Typically, a selective preference for protein is measured by direct observation of food choices over a short period (40). One possibility is that a shift in macronutrient prioritization is manifest as a “nudge” towards the selection of protein across all foods in the diet rather than a selective preference for specific foods that have high protein content. As we have already noted, food choice may be governed largely by habit and by a more general desire to consume alternative foods after a monotonous (low-protein) diet (41). Hence, observations of food intake may lack the sensitivity that is needed to detect subtle strategic changes in protein prioritization. If correct, our measure of protein valuation might be particularly useful alongside more traditional forms of assessment (31).

In summary, previous methods for assessing macronutrient intake have tended to rely on self-report (FFQ and diet diaries). Here, we approach the problem of quantifying macronutrient prioritization from a very different perspective—specifically, we introduce a method that quantifies and focuses on sensitivity to differences in macronutrient composition rather than overall macronutrient intake. Our novel methods capture aspects of behavior that are orthogonal to these traditional approaches, show excellent test-retest reliability, and are associated with a measure of muscle mass. We have highlighted areas where our approach might be refined, and we see exciting opportunities for its application, both in clinical and fundamental research.

## Supplementary Material

nxz124_Supplemental_File

## Notes

Funded by the Research Councils UK “Priming Food Partnerships” initiative supported by BBSRC, MRC, EPSRC, and ESRC (project reference BB/P023886/1).

Author disclosures: CMB, SA, BMC, MAG, AMJ, EJS, EAW and JMB, no conflicts of interest

Supplemental Table 1 is available from the “Supplementary data” link in the online posting of the article and from the same link in the online table of contents at https://academic.oup.com/jn/.

Abbreviations used: DEBQ, Dutch Eating Behavior Questionnaire; FFMI, fat-free mass index; VAS, visual analog scale.
