# Supplementary material for: Protein Valuation in Food Choice Is Positively Associated with Lean Mass in Older Adults
Source: J Nutr. 2019 Jun 14;149(11):2056–64. doi: 10.1093/jn/nxz124 (PMC6825819; doi:10.1093/jn/nxz124)
Supplement: nxz124_Supplemental_File [file nxz124_supplemental_file.pdf]

Supplementary data

Supplementary Table 1. Mean, confidence intervals, and *t*-test results (difference from 1) for odds ratios in Study 1 and Study 2.

	Study 1				Study 2			
	OR (95% CI)	*t*	*df*	*P*	OR (95% CI)	*t*	*df*	*P*
Protein	3.43 (2.67, 4.19)	6.35	167	<0.001	4.84 (2.37, 7.31)	3.09	89	0.002
Carbohydrate	3.37 (2.15, 4.59)	3.84	167	<0.001	2.24 (1.55, 2.94)	3.58	89	<0.001
Fat	0.96 (0.89, 1.03)	-1.17	167	0.241	1.32 (1.13, 1.50)	3.43	89	<0.001
Expected satiety	1.06 (1.01, 1.11)	2.50	167	0.013	1.40 (1.23, 1.57)	4.69	89	<0.001
Healthiness	1.00 (0.97, 1.04)	0.28	167	0.781	3.82 (2.69, 4.96)	4.94	89	<0.001
